# Usefulness of Novel Balloon‐Less Convex Probe Bronchoscope for Diagnosing and Staging of Lung Cancer via EBUS‐TBNA and EUS‐B‐FNA

**DOI:** 10.1002/rcr2.70506

**Published:** 2026-02-10

**Authors:** Yuki Takigawa, Ken Sato, Suzuka Matsuoka, Yuka Matsuo, Mayu Goda, Keisuke Shiraha, Hiromi Watanabe, Jun Nishimura, Kenichiro Kudo, Keiichi Fujiwara, Takuo Shibayama

**Affiliations:** ^1^ Department of Respiratory Medicine NHO Okayama Medical Center Okayama Japan

**Keywords:** balloon‐less convex‐probe endobronchial ultrasound bronchoscope, EBUS‐TBNA, EUS‐B‐FNA, lung cancer

## Abstract

In the winter of 2025, a novel balloon‐less convex‐probe endobronchial ultrasound bronchoscope (BLCP‐EBUS) (Fujifilm EB‐710US) was introduced in Japan. It features a balloon‐less design, flexible distal tip, improved angulation, and reduced outer diameter, though clinical experience remains limited. Here we report our initial experience using the EB‐710US for EBUS‐guided transbronchial needle aspiration (EBUS‐TBNA) and endoscopic ultrasound‐guided fine‐needle aspiration via the bronchus (EUS‐B‐FNA). An 86‐year‐old man with mediastinal lymphadenopathy underwent EBUS‐TBNA of a left lower paratracheal lymph node (station #4L). Adequate lesion contact and stable needle aspiration were achieved without a balloon, enabling successful tissue collection and adenocarcinoma diagnosis. A 73‐year‐old man required a re‐biopsy for a multiplex driver mutation test, and EUS‐B‐FNA of lymph node #8 was performed using a novel bronchoscope. Both procedures were safely completed under conscious sedation, with positive rapid on‐site cytology evaluation and sufficient tissue for adenocarcinoma diagnosis. The novel BLCP‐EBUS bronchoscope (EB‐710US) demonstrated favourable manoeuvrability and adequate lesion contact for both EBUS‐TBNA and EUS‐B‐FNA. The novel bronchoscope may enhance procedural safety, patient comfort, and diagnostic yield.

## Graphical abstract

In the winter of 2025, a novel balloon‐less convex‐probe endobronchial ultrasound bronchoscope (BLCP‐EBUS) (Fujifilm EB‐710US) was introduced in Japan. It features a balloon‐less design, flexible distal tip, improved angulation, and reduced outer diameter, though clinical experience remains limited. Here we report our initial experience using the EB‐710US for EBUS‐guided transbronchial needle aspiration and endoscopic ultrasound‐guided fine‐needle aspiration via the bronchus.

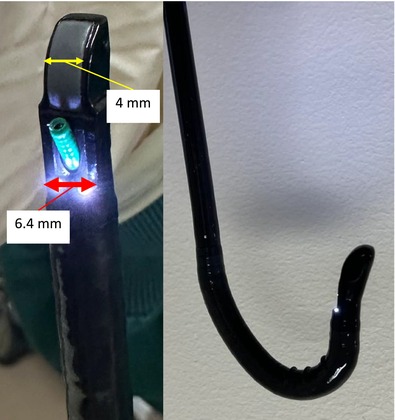

## 1 Introduction

Bronchoscopic diagnosis using endobronchial ultrasound (EBUS) has become increasingly important for accurate staging, tissue acquisition, and multiplex driver testing in lung cancer. Recently, as perioperative treatment strategies of non‐small cell lung cancer incorporating immune checkpoint inhibitors have been established, the clinical role of convex probe (CP)‐EBUS is expected to increase for lung cancer staging and diagnosing in more peripheral lymph node aspiration. In this context, the limitations of conventional CP‐EBUS systems—such as their relatively large outer diameter, limited manoeuvrability in distal airways, and dependence on balloon inflation for stable imaging—have prompted the development of a novel slim EBUS designed to enhance bronchial accessibility and diagnostic yield while preserving sufficient image quality.

**FIGURE 1:**
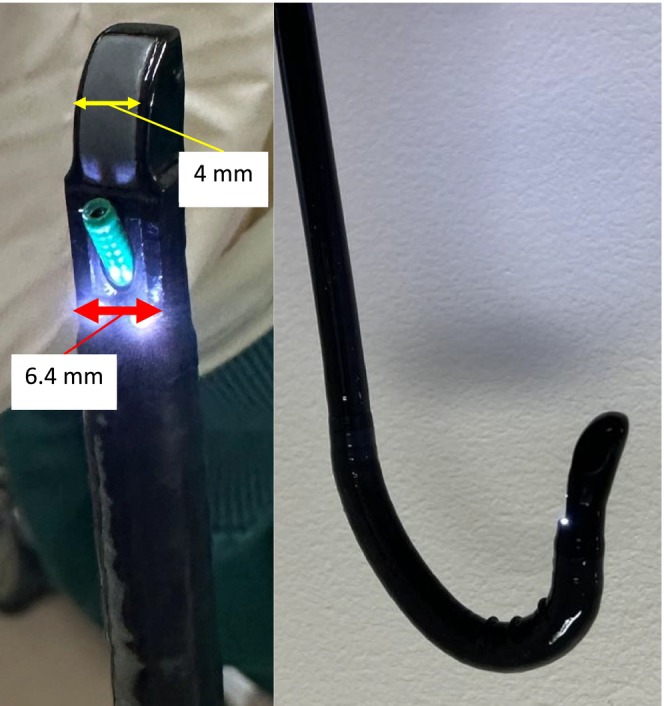
Balloon‐less convex‐probe EBUS (BLCP‐EBUS) bronchoscope (Fujifilm EB‐710US). Yellow line indicates approximately 4 mm; red line indicates 6.4 mm.

In the winter 2025, a balloon‐less convex‐probe endobronchial ultrasound (BLCP‐EBUS) bronchoscope (Fujifilm EB‐710US) became available for clinical use in Japan. This endoscope was specifically developed for bronchoscopic sample acquisition of lesions adjacent to the central airways and features a balloon‐less design without a distal rigid tip (spicule for anchoring the balloon), enhanced angulation capability, and a reduced outer diameter (Figure 1). To the best of our knowledge, there have been no previous reports describing the clinical experience with this bronchoscope in Japan. Here, we report our initial experience using the EB‐710US for EBUS‐guided transbronchial needle aspiration (EBUS‐TBNA) and endoscopic ultrasound‐guided fine‐needle aspiration via the bronchus (EUS‐B‐FNA). We present the first cases diagnosed and staged using this novel bronchoscope in December 2025.

## 2 Case Report

### 2.1 Case 1

**FIGURE 2:**
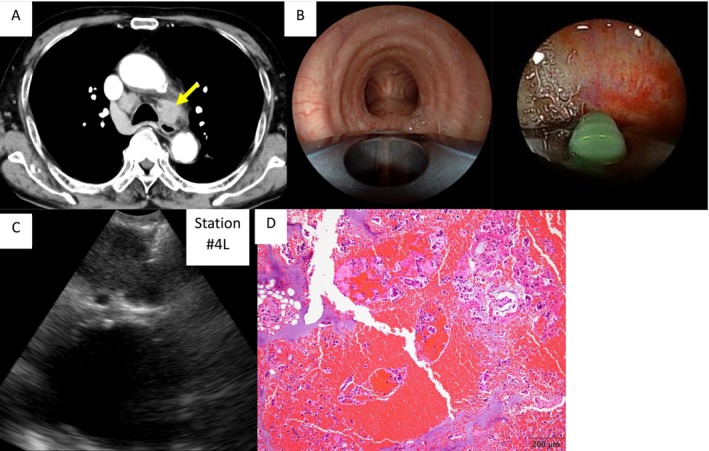
Chest computed tomography (CT), bronchoscope and pathological findings of Case 1. Chest computed tomography showing a #4L lymph node (LN) indicated by a yellow arrow. (B) Bronchoscopic images of the lower trachea (left) and during aspiration (right). (C) EBUS image showing aspiration at station #4L lymph node (LN). (D) Histopathological findings of adenocarcinoma.

An 86‐year‐old man presented to the otolaryngology department with hoarseness. Contrast‐enhanced computed tomography revealed mediastinal lymphadenopathy. Lung cancer was suspected, and a bronchoscopic examination was performed (Figure 2A). A BLCP‐EBUS bronchoscope (EB‐710US; Fujifilm) was inserted into the bronchus under conscious sedation using midazolam (2 mg) and pethidine (17.5 mg) as the initial doses. Additional 2% lidocaine spray and supplemental doses of midazolam and pethidine were administered as needed according to the severity of coughing or insufficient sedation. The target lesion was visualised through contact with the bronchial wall of the left lower trachea (Figure 2B). Adequate visualisation of the target lymph node (LN) station #4L was achieved, allowing stable needle aspiration (Figure 2C). Six needle aspirations were performed using a 22‐gauge TBNA needle, and rapid on‐site cytological evaluation (ROSE) was positive for malignant cells. The total procedure time was 42 min. Histopathological examination revealed an adenocarcinoma (Figure 2D). Based on the findings of positron emission tomography–computed tomography and brain MRI, the clinical stage was determined to be cT1cN3M1b(BRA) stage IVA. Gene panel testing revealed no detectable gene mutations and PD‐L1 expression rate was 5%. A single brain metastasis was detected in MRI, and treatment with immune checkpoint inhibitors was scheduled following radiotherapy for brain metastasis.

### 2.2 Case 2

**FIGURE 3:**
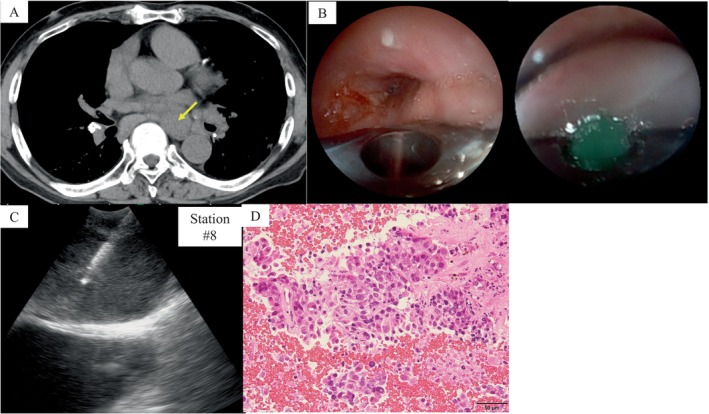
Computed tomography (CT), bronchoscope and pathological findings of Case 2. (A) CT showing a #8 lymph node (LN) indicated by a yellow arrow. (B) Bronchoscope images showing the oesophagus lumen (left) and during aspiration (right). (C) Endobronchial ultrasound bronchoscope (EBUS) image showing the aspiration of station #8 LN. (D) Histopathological findings indicating adenocarcinoma.

A 73‐year‐old man was diagnosed with lung cancer of the left lower lobe; however, the tissue specimen obtained was insufficient for driver mutation analysis, necessitating re‐biopsy. Sampling of LN #8 (Figure 3A) was required, and EUS‐B‐FNA was performed using a novel bronchoscope. Under conscious sedation with midazolam (2 mg) and fentanyl (0.02 mg), the BLCP‐EBUS bronchoscope (EB‐710US) was advanced into the oesophagus (Figure 3B). The lymph node was visualised via a transesophageal approach, and five needle passes were performed using a 22‐gauge needle, yielding ROSE‐positive results. Visualisation of the needle sheath and oesophageal lumen was adequate before and after the procedure (insufflation of air through the working channel with a 20‐mL syringe allowed visualisation of the oesophageal lumen; Figure 3C). The total procedure time was 22 min. Histopathological findings revealed squamous cell carcinoma (Figure 3D), and the clinical stage was cT2aN2bM0 stage IIIB. Gene panel testing revealed no detectable gene mutations, and PD‐L1 expression was negative (< 1%). The patient was being treated for idiopathic interstitial pneumonia, and treatment with carboplatin and paclitaxel was initiated as anticancer therapy.

## 3 Discussion

The novel slim BLCP‐EBUS bronchoscope, Fujifilm EB‐710US, was developed as a balloon‐less EBUS bronchoscope with a 2.2‐mm working channel and a distal outer diameter of 6.4 mm. Compared with previous endoscopes, the distal tip is smaller (approximately 4 mm) and the rigid portion is shorter, facilitating easier insertion. The direction of view was 10°, similar to that of a conventional flexible bronchoscope. Consequently, advancement into the trachea or oesophagus is technically easier than with a conventional bronchoscope, allowing smooth and trouble‐free insertion, even for operators with limited experience in bronchoscopy. The EB‐710US had a smaller profile than previously available convex‐probe bronchoscopes, and the maximum upward angulation was improved to 180 degrees (Figure 1). Certain mediastinal lymph node stations, such as the left lower paratracheal lymph nodes (stations #4L and #10L), are considered technically challenging for EBUS‐TBNA [1]. Despite initial concerns regarding probe–lesion contact in the absence of a balloon, the enhanced upward angulation enabled adequate contact, allowing six successful needle passes and sufficient tissue acquisition, as in Case 1.

The absence of a balloon may indeed represent a potential limitation in certain anatomical locations, particularly in mediastinal lymph node stations where unstable airway–probe contact and adjacent vascular structures warrant precise Doppler assessment (e.g., stations #4). In previous research [2] comparing aspiration for LN #4R, saline‐filled balloon use versus no balloon use in conventional CP‐EBUS, image quality was reported to be slightly inferior without balloon use; however, diagnostic yield, procedure time, and safety were not significantly different between the two approaches. However, a more recent study [3] demonstrated that a slim BLCP‐EBUS prototype (Fujifilm) used on human cadavers offers non‐inferior and potentially superior imaging, suggesting the potential for further reach into the lung periphery, enabling aspiration of the lymph nodes located in the inner and middle thirds of the lung.

We previously demonstrated the utility of a third‐generation endoscope (BF‐UC290F, Olympus) for EUS‐B‐FNA in *Respirology Case Reports*, which is attributable to its compact outer diameter (6.6 mm) and short rigid distal portion [4]. In this case, EUS‐B‐FNA using the novel BLCP‐EBUS bronchoscope was technically improved. Endoscopic visualisation of the oesophagus was clear, and tissue sampling was easily performed. Comfortable conscious sedation required only initial doses of midazolam and fentanyl without the need for additional administration, and the procedure was completed under stable hemodynamic conditions. The use of a BLCP‐EBUS bronchoscope may reduce patient discomfort owing to its small distal tip without spicule for anchoring the balloon. Moreover, pharyngeal and pyriform sinus mucosal injuries, including perforations, have been reported when using larger or rigid‐tipped probes. From a safety perspective, balloon‐less bronchoscopy without a rigid distal tip may therefore be advantageous [5].

Furthermore, the EB‐710US enhances the visibility of the aspiration needle. Unlike the conventional Olympus CP‐EBUS system, in which the needle sheath is located at the 2 o'clock position on the endoscopic view, the BLCP‐EBUS system used in this case (EB‐710US) has the needle sheath positioned at the 6 o'clock direction, which may affect procedural orientation. This characteristic may contribute to the improved procedural stability of recently adopted techniques for mediastinal lesions, such as EBUS‐guided intranodal forceps biopsy and EBUS‐guided cryobiopsy [6].

In our experience, the EB‐710US is useful for both transbronchial needle aspiration and transesophageal aspiration at the station. With the recent introduction of the thin convex‐probe EBUS bronchoscope [7] (BF‐UCP190F; Olympus), the range of EBUS devices for lesions adjacent to the airways has expanded to include more peripheral sites. Since BF‐UCP190F is compatible with only a 25‐gauge needle, appropriate bronchoscope device and needle size selection according to the clinical objective is essential.

This study has several limitations. As reports on the clinical use of EB‐710US are limited, further evaluation in larger cohorts is warranted. Further studies should assess aspiration success rates at individual lymph node stations.

In conclusion, because of its balloon less slim design and enhanced manoeuvrability, the novel BLCP‐EBUS bronchoscope appears to be a promising option for both EBUS‐TBNA and EUS‐B‐FNA procedures.

## Author Contributions

Yuki Takigawa and Ken Sato wrote the manuscript, which was reviewed by all co‐authors. All authors have approved the final version of the manuscript for submission.

## Funding

The authors have nothing to report.

## Consent

Written informed consent was obtained from the patient for publication of this manuscript and accompanying images, in accordance with journal requirements.

## Conflicts of Interest

The authors declare no conflicts of interest.

## Data Availability

The data that support the findings of this study are available from the corresponding author upon reasonable request.
